# Enhanced Sampling Molecular Dynamics Simulations Reveal Transport Mechanism of Glycoconjugate Drugs through GLUT1

**DOI:** 10.3390/ijms25105486

**Published:** 2024-05-17

**Authors:** Zhuo Liu, Xueting Cao, Zhenyu Ma, Limei Xu, Lushan Wang, Jian Li, Min Xiao, Xukai Jiang

**Affiliations:** 1 National Glycoengineering Research Center, Shandong University, Qingdao 266237, China; 2 State Key Laboratory of Microbial Technology, Shandong University, Qingdao 266237, China; 3 Biomedicine Discovery Institute, Monash University, Melbourne 3800, Australia

**Keywords:** GLUT1, glycoconjugate, anticancer drug, transport mechanism, molecular dynamics simulations

## Abstract

Glucose transporters GLUT1 belong to the major facilitator superfamily and are essential to human glucose uptake. The overexpression of GLUT1 in tumor cells designates it as a pivotal target for glycoconjugate anticancer drugs. However, the interaction mechanism of glycoconjugate drugs with GLUT1 remains largely unknown. Here, we employed all-atom molecular dynamics simulations, coupled to steered and umbrella sampling techniques, to examine the thermodynamics governing the transport of glucose and two glycoconjugate drugs (i.e., 6-D-glucose-conjugated methane sulfonate and 6-D-glucose chlorambucil) by GLUT1. We characterized the specific interactions between GLUT1 and substrates at different transport stages, including substrate recognition, transport, and releasing, and identified the key residues involved in these procedures. Importantly, our results described, for the first time, the free energy profiles of GLUT1-transporting glycoconjugate drugs, and demonstrated that H160 and W388 served as important gates to regulate their transport via GLUT1. These findings provide novel atomic-scale insights for understanding the transport mechanism of GLUT1, facilitating the discovery and rational design of GLUT1-targeted anticancer drugs.

## 1. Introduction

Cancer poses an urgent threat to human health globally [1,2,3]. A recent study predicts that approximately 27 million new cases will be diagnosed by 2030 [4]. Despite rapid growth in drug discovery in recent years, there remains a desperate need for novel, effective anticancer drugs to combat life-threatening cancer diseases [5]. In the 1920s, Warburg et al. revealed that tumor tissues typically obtain energy by metabolizing glucose into lactic acid at a rate nearly ten times higher than that of normal tissues [6]. This inefficient process necessitates tumor cells to acquire more glucose to meet their energy demands. Consequently, glucose-uptake-associated transporters are often highly expressed in tumor cells [7,8], potentially allowing for the development of transporter-targeted anticancer agents.

The major facilitator superfamily (MFS) is the largest superfamily of secondary active transporters found in both prokaryotes and eukaryotes [9]. MFS transporters are responsible for transporting diverse substrates, including nutrients, neurotransmitters, and ions. Glucose transporters (GLUTs) belong to the SLC2A family, which is a member of the group of MFS transporters (16 families in total). Mueckler et al. firstly purified and functionally characterized GLUT1 experimentally, and the crystal structure of its inward-open conformation in human has been determined using crystallization method [10,11]. GLUT1 features a typical topological fold comprising 12 conserved transmembrane helixes, divided into the N-terminal domain (helices 1–6) and C-terminal domain (helices 7–12). Studies have shown that GLUT1 is often highly expressed in cells with a high demand for glucose, including red blood cells, brain endothelial cells, and tumor cells [7,8,12,13]. GLUT1 deficiency (GLUT1DS) can cause early-onset seizures, microcephaly, and impaired development [14,15]. As abnormal glycometabolism in tumor cells is believed to be inherently related to the overexpression of GLUT1, the expression level of the transporter was taken as an important prognostic indicator of cancer occurrence in the clinic research [16]. Also, it is believed that GLUT1 can be a promising therapeutic target for the discovery of anticancer drugs [17,18,19].

Glycoconjugate anticancer drugs targeting GLUT1 demonstrate improved delivery efficacy to tumor tissues [20,21]. Notably, GLUT1-targeted drugs may function via two different modes of action. For example, 6-D-glucose-conjugated chlorambucil binds tightly to GLUT1, inhibiting its transport function [22]. In contrast, 6-D-glucose-conjugated methane sulfonate enters the cell via GLUT1 transport to act on its intracellular target [23]. While glycoconjugates are believed to be a valuable resource in the discovery of GLUT1-targeted anticancer drugs, glycoconjugate screening largely relies on trial-and-error experiments. Additionally, the mechanism by which GLUT1 interacts with its substrates remains elusive, significantly hindering the rational design of anticancer drugs targeting GLUT1.

Molecular dynamics (MD) simulations provide a powerful tool to examine the structural dynamics of biomacromolecules. Compared to experimental approaches, MD simulations enable the exploration of protein motions and protein–ligand interactions at high spatiotemporal resolutions [24]. Therefore, this method has been widely used in studies of protein function mechanisms, especially membrane proteins, including transporters, receptors, and ion channels [25,26,27,28].

Here, we employed all-atom MD simulations to explore the transport mechanism of GLUT1 using three ligands: its natural substrate (glucose) and two glycoconjugates (6-D-glucose-conjugated methane sulfonate and 6-D-glucose-conjugated chlorambucil). Through steered molecular dynamics simulations and umbrella sampling techniques, we elucidated the thermodynamic pathways that govern the transport of each substrate through GLUT1. Importantly, their transport processes were analyzed comparatively to identify the key determinants of the transport function of GLUT1, which is typically difficult to achieve using conventional biochemical and biophysical experimental methods. We identified the key interactions and residues that were associated with regulation of the substrate recognition, binding, translocation, and release. These findings provide atomic-scale insights into the interaction between GLUT1 and different substrates, facilitating further studies on the discovery of GLUT1-targeted anticancer drugs.

## 2. Results and Discussion

### 2.1. Conformational Analysis of GLUT1 during Transport

**Figure 1:**
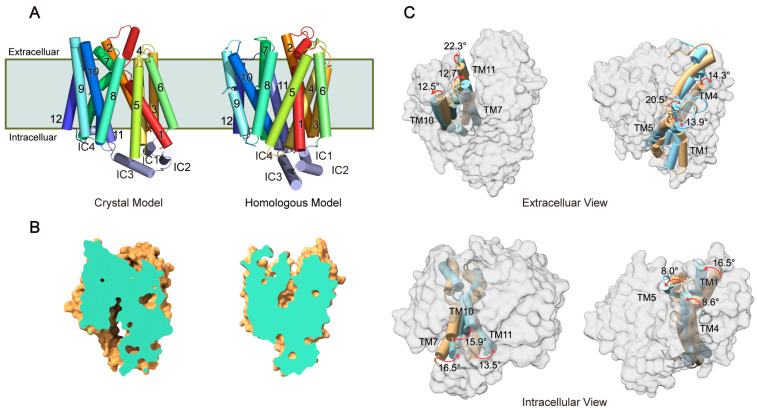
Representative conformations of human GLUT1. (**A**) Crystal structural model (PDB ID: 4PYP) and homologous structural model of GLUT1. The indices of the secondary structures are labeled. The membrane is depicted using a light green shadow. (**B**) Cross sections of GLUT1 at its inwardly and outwardly opening states. (**C**) Structural superposition of outwardly (yellow) and inwardly (blue) opening conformations shows the structural arrangement of GLUT1. The helices forming the transport channel are depicted as cartoon models, while the remaining are depicted as surface models. The rotation degrees of individual helices are labeled.

As with many MFS transporters, GLUT1 undergoes a conformational transition during transport, transitioning from an outwardly opening state to an inwardly opening state [29]. Notably, the outwardly opening conformation participates in substrate recognition and binding, while the inwardly opening conformation primarily contributes to the release of the substrate into the cytoplasm. Prior to this study, only the inwardly opening conformation of human GLUT1 had been determined experimentally (PDB ID: 4PYP, Figure 1A) [10]. To fully understand the complete transport mechanism, the outwardly opening conformation of GLUT1 needs to be determined. The bacterial homolog (XylE from *Escherichia coli*, PDB ID: 4GBZ) is the only MFS member for which crystal structures of different conformations have been obtained, including the outwardly opening conformation [30]. Using a homology-modeling algorithm, we developed an outwardly opening model of GLUT1 (Figure 1A) using XylE as the structural template [31].

The transmembrane segments of GLUT1 comprise 12 helices (TM1–TM12), each divided into an N-terminal domain (TM1–TM6) and C-terminal domain (TM7–TM12), with the intracellular segments consisting of four helical bundles (IC1–IC4). Structural analysis revealed that the conformational transition of GLUT1 significantly changed the accessibility of its transport channel (Figure 1B). Specifically, the GLUT1 channel was exposed to the intracellular and extracellular environments in the inwardly opening and outwardly opening states, respectively. The conformational transition can be characterized by the movement of several transmembrane helices (Figure 1C). By comparing the structures at two different states, we found that TM1, TM4, and TM5 in the N-terminal domain and TM7, TM10, and TM11 in the C-terminal domain experienced structural rearrangement to varying degrees during the conformational transition. Additionally, it was precisely these helixes that formed the transport channel of GLUT1.

### 2.2. The Overall Pathway of Glucose Transport through GLUT1

**Figure 2:**
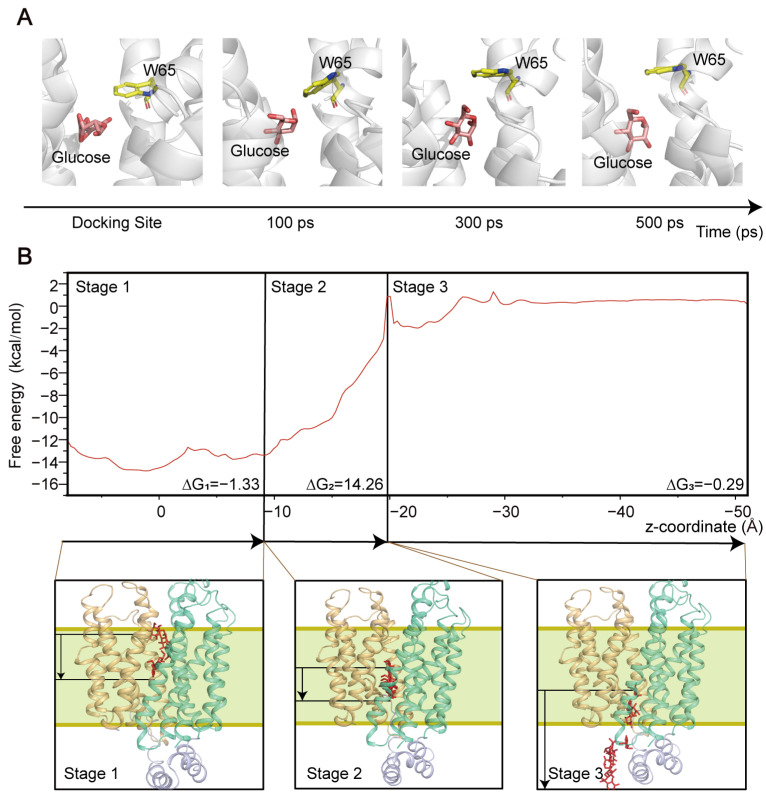
Transport pathway of glucose through GLUT1. (**A**) The configurations of the glucose located at the entrance site of GLUT1 during the unbiased MD simulations. (**B**) Free energy profile for glucose transport through GLUT1. The structural snapshots depicting the transport process show the glucose molecules represented as red stick models.

To understand the overall transport mechanism of GLUT1, we first investigated the interactions of glucose, the prototypical substrate. In the initial GLUT1–glucose complex generated using molecular docking, the glucose molecule was located at the entrance site of the GLUT1 recognition pocket. Unbiased MD simulations showed that the glucose molecule remained stable in this pocket (Figure 2A), indicating that the docking site closely aligned with the substrate recognition pocket, thereby allowing for stable binding of the glucose. Interestingly, we found that the residue W65 on TM2 adjusted its conformation upon glucose binding, orientating its side chain (the indole ring) approximately 90° towards the channel cavity, which impeded the escape of the glucose molecule (Figure 2A). These findings suggest that W65 is likely an important extracellular gate for modulating glucose recognition and binding to GLUT1, which is consistent with a previous study [32].

Due to the limited sampling capability of unbiased MD simulations, it is almost impossible to observe the spontaneous transport of ligands through GLUT1 within simulation-accessible timescales. Therefore, we employed steered MD simulations to construct the entire transport trajectory from the recognition site to the intracellular environment. This method has been widely used in the study of transport mechanisms of diverse transporters [33,34,35]. After MD simulation, TM1, TM4, and TM11, which construct the transport channel of GLUT1, were slightly inclined, indicating the tendency of conformational transition of GLUT1 (Figure S1). We then performed umbrella sampling simulations to examine the thermodynamics governing glucose transport via GLUT1. Based on the free energy profile, the entire transport process could be divided into three continuous stages (Figure 2B). In stage 1, the glucose molecule moved from the recognition site to the central binding pocket of GLUT1. During this stage, the free energy profile remained relatively steady, with an overall free energy change of −1.33 kcal/mol (∆G_1_), indicating an energy-favorable process. In stage 2, the glucose molecule traversed the central binding pocket, during which the free energy profile climbed sharply, with a free energy barrier of 14.26 kcal/mol (∆G_2_). In stage 3, the glucose molecule gradually exited the channel and entered the intracellular space. During this stage, the free energy slightly decreased (by 0.29 kcal/mol; ∆G_3_). Previous studies have revealed that the structural conformation of the central binding pocket is part of the GLUT1′s transition [36,37], which is necessary for the substrate transport. Our computational results indicate that passage through the central binding pocket was the most energy-consuming period during transport. Collectively, these results suggest that the interactions built within the central binding pocket may play a determinant role in regulating the transport function of GLUT1.

### 2.3. Interaction Dynamics between GLUT1 and Glucose during Transport

**Figure 3:**
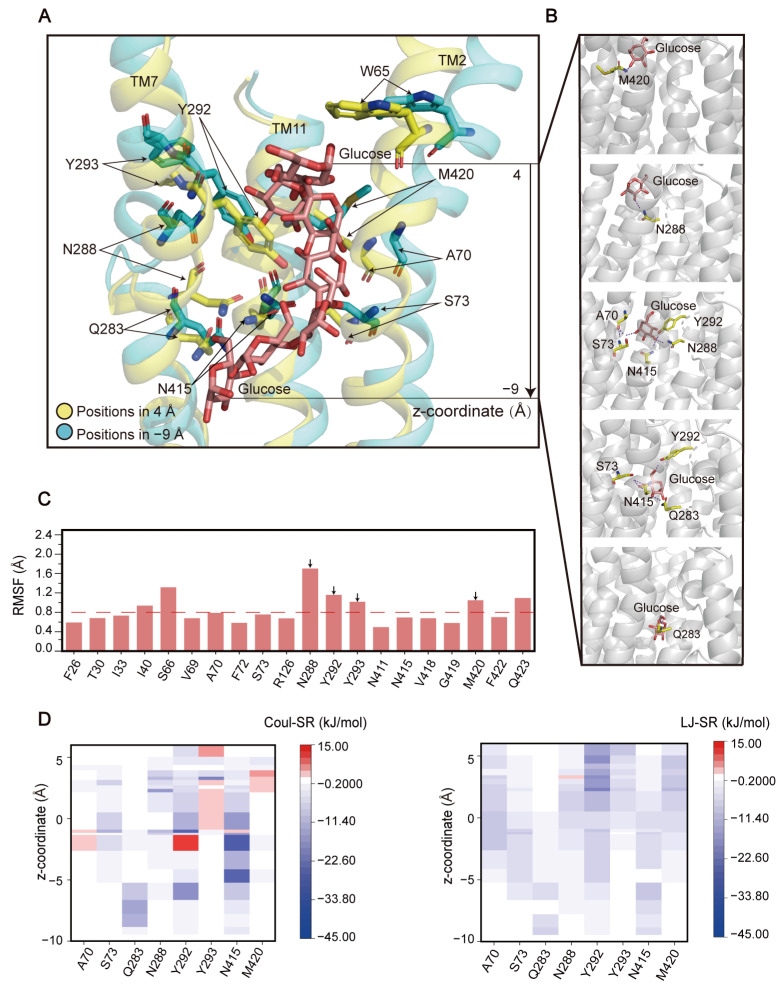
Interaction between glucose and the recognition pocket of GLUT1. (**A**) The movement trajectory of the glucose molecule. The key residues that interact with glucose are depicted as stick models at the starting and ending time points. (**B**) Interaction networks between glucose and GLUT1. Hydrogen bonds are depicted by dashed lines. (**C**) Root mean square fluctuations (RMSFs) of backbone atoms of the residues in the recognition pocket. The dashed line indicates a cutoff level of RMSF, and the residues contributing to key interactions are labeled with black arrows. (**D**) Polar (Coul-SR) and hydrophobic (LJ-SR) interactions formed between the key residues and the glucose molecule.

As described above, during the transport of glucose by GLUT1, the glucose molecule initially bound to the recognition pocket of GLUT1, then entered the central binding pocket before finally being released from the exit site. The conformational transition of GLUT1 is considered a prerequisite for its transport function [38]. In the MD simulations, we observed that GLUT1 adopted various conformations at both the residue and secondary structure levels. To detail the transport process, we examined the interactions between GLUT1 and glucose during each transport stage.

(**1**) **Passing through the recognition pocket.** The glucose molecule gradually entered the GLUT1 channel, with the Z-coordinate decreasing from 4.3 Å to −9.2 Å (Figure 3A). The detailed interactions between the glucose molecule and the recognition pocket are shown in Figure 3B. Initially, the hydroxyl groups at C2 and C3 of the glucose formed hydrogen bonds with M420 and N288, respectively. The glucose molecule then established an extensive interaction network involving A70, S73, N288, Y292, and N415. Finally, the glucose molecule left the recognition pocket due to attraction to Q283. Through visualizing the structural snapshots of the GLUT1-glucose complex, we found that Q283, N288, Y292, and Y293 on TM7, as well as M415 on TM11, oscillated up and down as the translocation of glucose in the binding pocket occurred (Figure 3A). Calculation of the root mean square fluctuations (RMSFs) revealed that these residues remained structurally flexible during the passing of glucose through the recognition pocket (Figure 3C).

**Figure 4:**
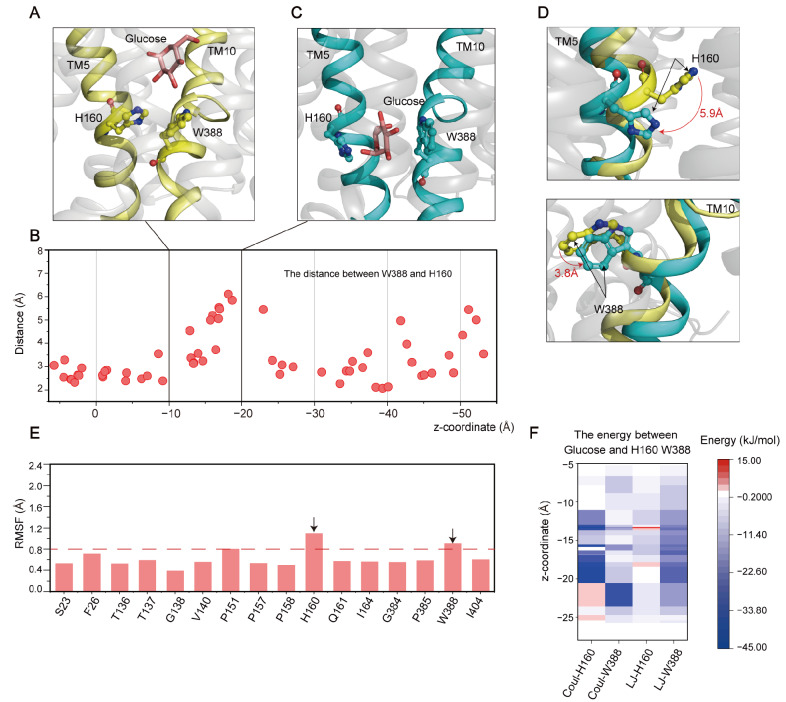
Interaction between glucose and the central binding pocket of GLUT1. (**A**) Structural snapshot of the glucose molecule reaching the extracellular side of the H160-W388 gate. (**B**) Distances between H160 and W388 residues. (**C**) Structural snapshot of the glucose molecule passing the H160-W388 gate. (**D**) Conformational rotations of H160 and W388. The red arrows represent the direction of the residue fluctuation. (**E**) RMSF of the backbone atoms of the residues in the central binding pocket. (**F**) Polar (Coul-SR) and hydrophobic (LJ-SR) interactions formed between the gated residues and glucose.

Subsequently, the electrostatic and hydrophobic interactions between each residue and the glucose molecule were quantified (Figure 3D). N288, Y292, Y293, and N415 dominated the interactions with glucose. Interestingly, A70, N288, Y292, Y293, N415, and M420 made repulsive interactions with the glucose at various periods. The extensive interactions of these residues and their flexible movements may drive the translocation of the glucose molecule into the next stage (i.e., entering the central binding pocket). Previous studies have demonstrated that Y292 and Y293 were involved in the closure of the extracellular gate and the conformational transition of GLUT1 [39,40]. Mutations in N415 and M420 have an impact on the specificity of substrate recognition, although they do not affect GLUT1 transport activity [41]. Crystallography studies have also shown that N288 and Q283 form hydrogen bonds with glucose [10,37], although mutations at these single sites do not completely disrupt GLUT1 function [42]. This can be interpreted through our simulation findings, which implied that the recognition and binding of glucose, the closure of the extracellular gate, and the induction of conformational changes were likely controlled by multiple residues that cooperatively facilitate the translocation of glucose into the central binding pocket.

(**2**) **Passing through the central binding pocket.** After passing through the recognition site, glucose enters the central binding pocket. A previous study proposed that W388 on TM10 is important for glucose to pass through the central binding site via an unknown mechanism [43]. In our simulations, we observed that H160 and W388 together constituted a checkpoint to seal the GLUT1 transport channel (Figure 4A). Interestingly, the distance between these two residues could transiently increase from ~3 Å to ~6 Å (Figure 4B), thereby ensuring that the glucose molecule (size: ~5.2 Å) could pass (Figure 4C). Further analysis revealed the counterclockwise rotation of the imidazole ring of H160 by 5.9 Å and the clockwise rotation of the indole ring of W388 by 3.8 Å (Figure 4D), indicating the opening of the GLUT1 channel. TM5 and TM10 also expanded outward upon the conformational changes of H160 and W388 (Figure 4D). RSMF calculations revealed that H160 and W388 were quite flexible (Figure 4E), which may enable their conformational transition and regulatory roles during transport. Energy analysis showed that H160 formed strong polar interactions with glucose and W388 made extensive hydrophobic interactions with glucose as it passed through the central binding pocket (Figure 4F). Given that the glucose molecule must overcome the highest free energy barrier when passing through the central binding pocket (Figure 2B), these results suggest that passing through the H160 and W388 gate is a key obstacle for glucose transport.

**Figure 5:**
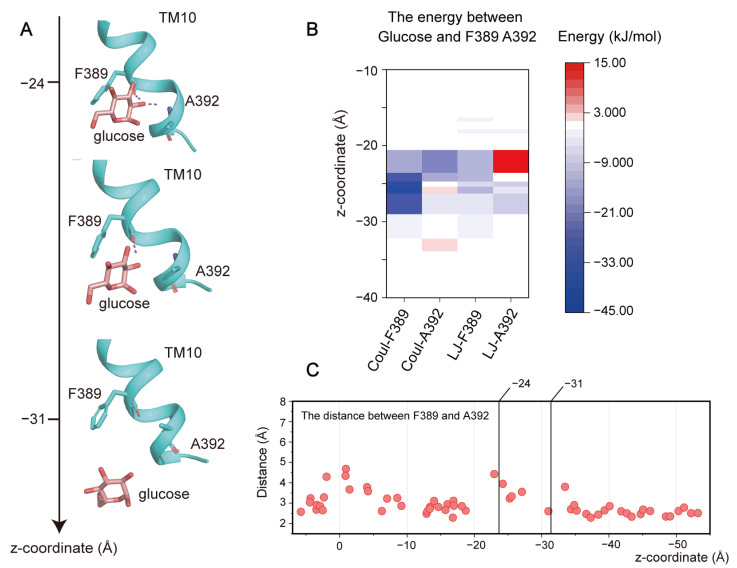
Interaction between glucose and the exit site of GLUT1. (**A**) Structural snapshot of glucose passing the exit site. (**B**) Polar (Coul-SR) and hydrophobic (LJ-SR) interactions formed between F389, A382, and glucose. (**C**) Distances between F389 and A392.

Interestingly, when MD simulations were undertaken with an inwardly open GLUT1 as the initial structure, the glucose easily passed through the gate formed by H160 and W388 (Figure S2). A previous study involving XyIE (a homolog of human GLUT1) revealed that the movement of TM10 helix where W388 is located initiated its conformational transition and eventually promoted the release of the substrate [30]. These results indicate that H160 and W388 mediate the conformational transition of GLUT1 and are key elements in regulating the transport function.

(**3**) **Leaving from the exit site.** After passing through the central binding pocket, glucose prepares to leave the exit site and enter the intracellular space. During this process, glucose primarily formed interactions with F389 and A392 (Figure 5A). Interaction energy analysis revealed that a polar interaction with F389 attracted glucose towards the intracellular side, while a repulsive interaction from A392 possibly assisted in releasing glucose from GLUT1 (Figure 5B). During this stage, only minimal changes in the conformations of both residues were observed, with the distance between them remaining relatively constant (Figure 5C). This is consistent with a previous study that reported only a small number of interactions between the substrate and GLUT1 during the releasing stage [10]. When combined with the free energy analysis, these results indicate that the release of the substrate was likely a spontaneous and energy-favorable process.

### 2.4. Transport of Glycoconjugate Drugs through GLUT1

**Figure 6:**
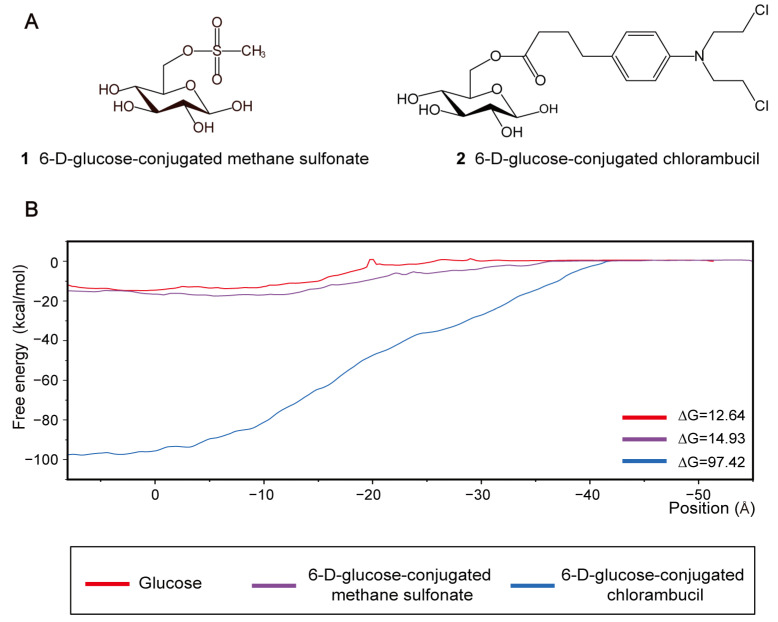
Transport free energy analysis of two glycoconjugate drugs. (**A**) Chemical structures of 6-D-glucose-conjugated methane sulfonate and 6-D-glucose-conjugated chlorambucil. (**B**) Free energy profile for the glycoconjugates and glucose transport through GLUT1.

**Figure 7:**
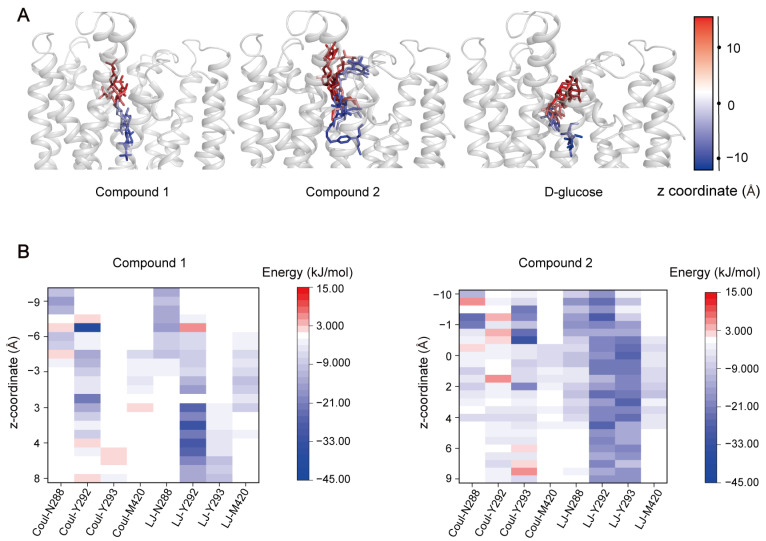
Comparative analysis of two glycoconjugate drugs and glucose in the recognition pocket. (**A**) The movement trajectory of substrates during passing through the recognition pocket. The color spectrum indicates the z-coordinates of the mass center of the substrates. (**B**) Polar (Coul-SR) and hydrophobic (LJ-SR) interactions were formed between key residues and the two compounds.

**Figure 8:**
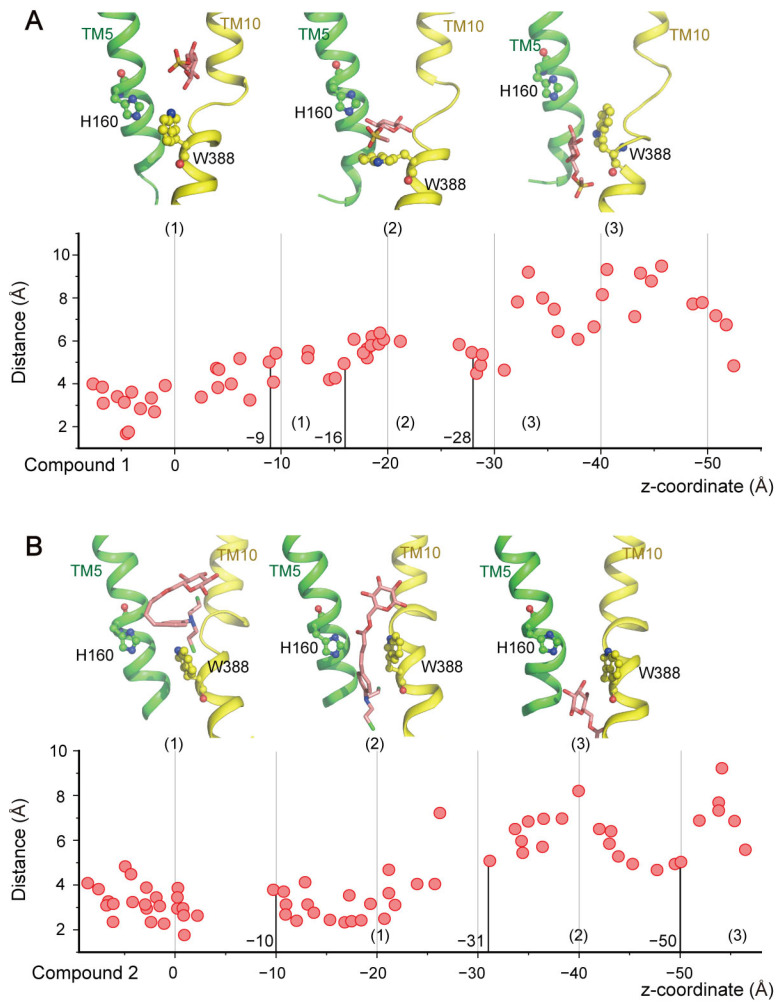
Interaction between the two glycoconjugate drugs and the central binding pocket of GLUT1. Distances between H160 and W388. Structural snapshots of the substrate describing the transport process are shown, with the substrate shown as a pink stick model. (**A**) Compound **1** and (**B**) Compound **2**.

**Figure 9:**
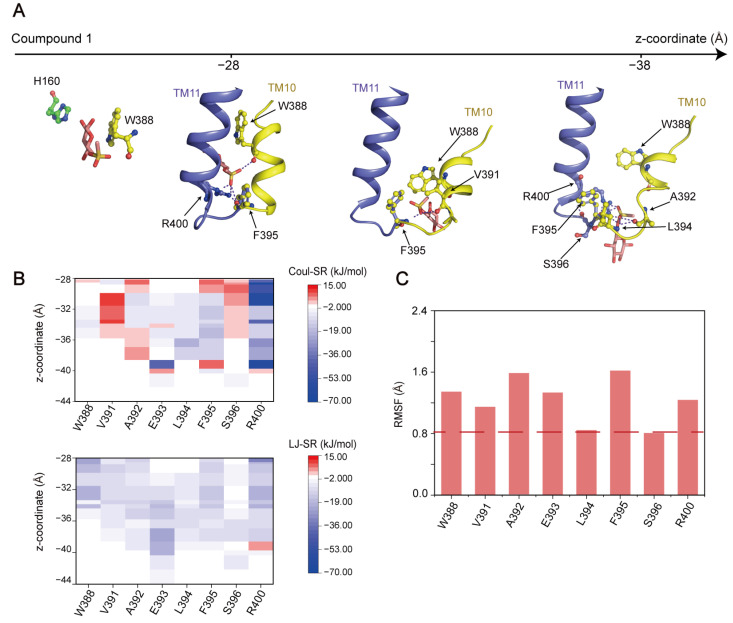
Interaction between Compound **1** and the exit site of GLUT1. (**A**) Structural snapshots of Compound **1** describing the transport process. The substrate is shown as a pink stick model. (**B**) Polar (Coul-SR) and hydrophobic (LJ-SR) interactions between the substrate and interacting residues. (**C**) RMSFs of the backbone atoms of the interacted residues around the exit site.

Examination of the structure–function relationship of protein targets has played an increasing role in drug discovery and development [44]. Targeting GLUT1, which is overexpressed in tumor cells, glycoconjugates are considered promising candidates in the development of new-generation anticancer drugs [45,46]. Therefore, we examined the interactions between GLUT1 and two compounds: 6-glucose-conjugated methane sulfonate (Compound **1**) and 6-glucose-conjugated chlorambucil (Compound **2**) (Figure 6A). Using steered and umbrella sampling simulations, we characterized the free energy profiles of these glycoconjugates (Figure 6B). The profile of Compound **1** closely resembled that of glucose, with an overall free energy barrier for transport of 14.93 kcal/mol (compared to glucose’s overall free energy barrier of 12.64 kcal/mol). In contrast, the profile of Compound **2** differed significantly from that of the glucose, exhibiting a substantially higher overall free energy barrier of 97.42 kcal/mol. These results suggest that Compound **1** is likely a transportable substrate of GLUT1, while Compound **2** is not. In the following sections, we delve into the mechanisms underlying these differences in terms of their interactions with GLUT1.

(**1**) **Compounds 1 and 2 showed different binding behaviors in the recognition pocket.** While Compound **1** smoothly traversed the recognition pocket within the simulations, resembling the behavior of glucose, Compound **2** could not pass this pocket. Instead, it adopted a unique folded conformation within it (Figure 7A). We further calculated the interaction energy of M288, Y292, Y293, and M420 with the substrates. These four residues play a significant role in the transport of glucose (Figure 3D). As expected, the interactions between these residues and Compound **1** evolved over time (Figure 7B), representing the dynamic process of transport. In contrast, Compound **2** formed steady interactions, most notably with Y292 and Y293 (Figure 7B). These results suggest that Compound **2** may inhibit transport by GLUT1 by competitively binding to its recognition pocket.

(**2**) **H160-W388 formed a gate that regulated the transport of glycoconjugate drugs.** As H160 and W388 served as a gate, constituting the highest free energy barrier during glucose transport, we analyzed the interactions between glycoconjugate drugs and GLUT1 when passing through this gate. For clarity, we defined three periods for this process: pre-pass (1), passing (2), and post-pass (3). In the Compound **1** system (Figure 8A), the distance between H160 and W388 exhibited minor fluctuations around 4 Å. Then, the distance increased up to 6.3 Å in the passing period, allowing for the translocation of Compound **1**. Interestingly, the distance continued to increase in the final post-passing period, likely due to conformational rearrangement of GLUT1 following transport. Generally, the dynamic behavior of Compound **1** was similar to that of glucose. However, this was not the case with Compound **2** (Figure 8B). In that system, the time in the pre-pass period (2 ns) was substantially longer than that of Compound **1** (0.7 ns), with the distance between H160 and W388 increasing up to 8.2 Å in the passing period. Notably, Compound **2** was required to form an extended conformation to cross the H160-W388 gate. These findings reveal that Compound **2** can only function as a competitive inhibitor, but not a transport substrate because it could not pass the central binding pocket of GLUT1 and therefore could not be transported by GLUT1. As observed with glucose, these results further demonstrated the critical roles of H160 and W388 in controlling substrate transport in GLUT1.

(**3**) **Compound 1 was released from GLUT1 due to repulsive interactions.** After traversing the H160-W388 gate, Compound **1** was ready to be released from GLUT1 and enter the cytoplasm. Analysis of the interactions between Compound **1** and the exit site of GLUT1 showed that Compound **1** had interactions with residues V391, L392, L394, and S396 on TM10, and R400 on TM11 (Figure 9A). Interestingly, only residue R400 formed attractive interactions with Compound **1** during this stage, especially the first half (Figure 9B), which promoted the substrate to move downward and approach the exit site of GLUT1. In contrast, the other residues, including V391, A392, F395, and S396, primarily made repulsive interactions with Compound **1** (Figure 9B). RMSF analysis revealed that the conformations of R400, V391, A392, and F395 were more flexible than those of the other residues in the GLUT1 exit site (Figure 9C), and were located near the intracellular side of GLUT1. Therefore, we proposed that these flexible residues on TM10 and TM11 may function as a releasing pocket. When the compound went through the central binding pocket through W388, their repulsive interactions promoted the release of Compound **1** from GLUT1. As is consistent with the glucose observations (Figure 5), these results indicate that the release of the substrate was primarily driven by the repulsive interactions with residues located on TM10 of GLUT1.

## 3. Materials and Methods

### 3.1. Structural Models of GLUT1

The experimentally determined structure of human GLUT1 showed an inwardly opening conformation [10]. To examine the substrate recognition and binding processes, we developed structural models of human GLUT1 in the outwardly opening conformation using the homology modeling method, which is one of the most reasonable and widely used conformational analysis methods. The crystal structure of GLUT1 homology from *Escherichia coli* (PDB ID:4GBZ) was used as the outward-open structural template [31]. MODELLER was used to construct 100 outwardly opening models using default parameters [47]. The rationality was evaluated through Ramachandran diagrams generated in PROCHECK (http://www.ebi.ac.uk/thornton-srv/databases/pdbsum/ accessed on 26 April 2023) [48].

### 3.2. Ligand Structure and Topology

The chemical structures of glucose, 6-D-glucose-conjugated chlorambucil, and 6-D-glucose-conjugated methane sulfonate were drawn in ChemBioDraw 14 and converted to three-dimensional models in ChemBio3D Ultra 14.0.0.117. Energy minimization was undertaken to alleviate any potential intramolecular steric clashes. The topological parameters for the substrates were generated using the SwissParam server (http://swissparam.ch/ accessed on 5 May 2023) according to the standards of the CHARMM force field [49].

### 3.3. Molecular Docking

Molecular docking can help us understand the binding patterns between small molecules and proteins. Here, we performed molecular docking to prepare the initial configurations for the following MD simulations. The outwardly opening GLUT1 structure was used in the docking analysis. AutoDock Tools 1.5.6 was used to prepare the docking parameter files, wherein polar hydrogen atoms were added and gasteiger charges were assigned by default [50]. The grid box was adjusted according to the docking site, while other parameters were set based on previous studies [51,52]. The docking structure with the lowest energy (i.e., the best binding score) was used for further MD simulations.

### 3.4. System Preparation for MD Simulations

The structural models of protein–ligands embedded in the membrane were constructed using the CHARMM-GUI membrane builder (https://charmm-gui.org/?doc=input/membrane.bilayer accessed on 10 May 2023) [53]. According to the literature, the phospholipid bilayer was fully composed of phosphatidylcholine [32]. In detail, 80% didecanoyl phosphatidylcholine (DCPC) and 20% dilauroyl phosphatidylcholine (DLPC) were employed. Previous studies revealed that such phospholipids with shorter fatty acyl chains can improve the sampling of the membrane-protein simulation system [54]. The dimensions of the bilayer in the x-y plane were 10 × 10 nm^2^, which contained 216 DCPC and 54 DLPC molecules. GLUT1 was placed at the center of the membrane. The simulation system was set to be a cubic box, and TIP3P water models were used to solvate the system. Additionally, 65 Na^+^ and 73 Cl^−^ atoms were added for system neutralization. In total, there were over 110,000 atoms in each simulation system.

### 3.5. Steered MD Simulations

Steered simulations are often used for an enhanced sampling approach in the research of biomolecular systems [55,56,57]. We utilized them to build the transport pathway for substrates to travel through GLUT1. To enable the smooth movement of substrates, a harmonic potential was applied between the mass centers of substrates and GLUT1 along the *Z*-axis [58,59]. The initial force constant of the potential was set to 500 kJ/mol/nm^2^, and the pulling velocity was set to 1 nm/ns. Other simulation settings were introduced in the simulation parameters section below.

### 3.6. Free Energy Analysis

Umbrella sampling simulations were performed to calculate the free energy profiles of GLUT1 transporting different substrates [55,56,57]. Initially, structural snaps from the steered simulations were generated with intervals of 0.2 nm, producing 30 simulation windows for each system. In each window, the center of mass of the substrate was harmonically restrained at various fixed *Z*-axis coordinates. Each window was simulated for 30 ns, resulting in a total of 900 ns of all-atom trajectories used to analyze a single free energy profile. The free energy was calculated using the weighted histogram analysis method (WHAM) [60].

### 3.7. Simulation Parameters

All simulations were performed using Gromacs 2019 with the CHARMM36 force field [61,62]. Energy minimization was performed to achieve a force maximum below 1000 kJ/mol/nm^2^, followed by equilibration simulations conducted using a six-step procedure as described previously [63]. The Particle Mesh Ewald method [64] and Lennard–Jones potential algorithm [65] were used to calculate the electrostatic and van der Waals interactions, respectively. V-rescale was used to maintain the system temperature at 310 K, and the semi-isotropic coupling algorithm with the Parrinello–Rahman barostat was used to maintain the pressure at 1 bar [66,67,68]. The time step was 2 fs, and the trajectory was recorded every 10 ps.

### 3.8. Data Analysis and Visualization

The biophysical quantities were analyzed using the internal tools of GROMACS 2019. All analytical charts, including line graphs, bar charts, and heat maps, were generated using the Origin 2022 program. The molecular structure was visualized using PyMOL 2.5.5.

## 4. Conclusions

**Figure 10:**
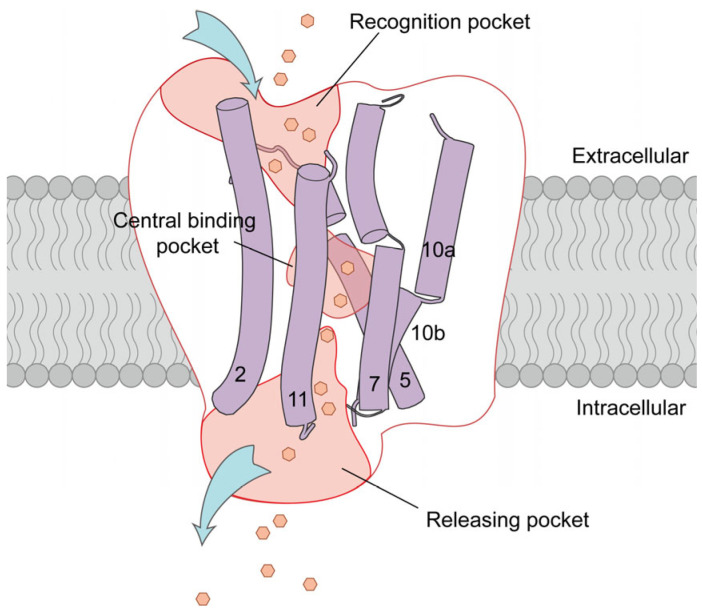
Transport model of GLUT1. The three transport pockets identified in this study are highlighted. The key helixes that constructed the transport channel are labeled with purple tubes. The substrates are indicated by hexagons.

Cancer cells require large quantities of glucose due to inefficient energy production through anaerobic glycolysis. Consequently, increased GLUT1 expression has been observed in various cancer types, including lung cancer, breast cancer, and bladder cancer [69,70,71,72], indicating GLUT1′s significance as a prognostic indicator for tumorigenesis and as a promising target for the discovery of novel anticancer drugs. In the present study, we employed glucose, the natural substrate of GLUT1, to investigate the thermodynamic mechanisms underlying the transport. The continuous transport process was defined (Figure 10). The detailed transport dynamics and key residues in GLUT1 responsible for the interaction with the glucose were identified, including the key residues (N288, Y292, Y293, M420) functioning in the recognition pocket and the H160-W388 gating site that regulated the substrate transport. Importantly, the transport processes of two glycoconjugate drugs via GLUT1 were investigated for the first time. Our results revealed that 6-substituted glycoconjugates, exemplified by methane sulfonate, may enhance drug targeting to GLUT1, providing potential avenues for targeted transport through GLUT1 in further research. In contrast, the 6-substituted glycoconjugates of dialkylating antitumor compound chlorambucil were more likely to function as specific inhibitors of GLUT1. These results enhance our understanding of the transport mechanism employed by human GLUT1 and provide a structural template for understanding the mechanisms of GLUT1 from different species. Further, our study also provides valuable information for the rational design of novel GLUT1-targeting drugs to address the problem of cancer [73,74].

## Data Availability

All data are included in the main text and Supplementary Materials.

## Supplementary Materials

The following supporting information can be downloaded at: https://www.mdpi.com/article/10.3390/ijms25105486/s1.
