# Supplementary material for: Enhanced Sampling Molecular Dynamics Simulations Reveal Transport Mechanism of Glycoconjugate Drugs through GLUT1
Source: Int J Mol Sci. 2024 May 17;25(10):5486. doi: 10.3390/ijms25105486 (PMC11122603; doi:10.3390/ijms25105486)
Supplement: Supplementary file 1 [file ijms-25-05486-s001.zip › ijms-2993309-supplementary.pdf]

Supporting information

Enhanced Sampling Molecular Dynamics Simulations Reveal Transport Mechanism of Glycoconjugate Drugs through GLUT1

Zhuo Liu[1], Xueting Cao[1], Zhenyu Ma[1], Limei Xu[2], Lushan Wang[2], Jian Li[3], Min Xiao[1], Xukai Jiang[1]*

[1] National Glycoengineering Research Center, Shandong University, Qingdao 266237, China;

[2] State Key Laboratory of Microbial Technology, Shandong University, Qingdao 266237, China;

[3] Biomedicine Discovery Institute, Monash University, Melbourne 3800, Australia.

Running title: Transport of glycoconjugate drugs by GLUT1

***Correspondence:**

Xukai Jiang, K6-229, Binhai Road 72, Shandong University Qingdao Campus.

Email: xukai.jiang@sdu.edu.cn

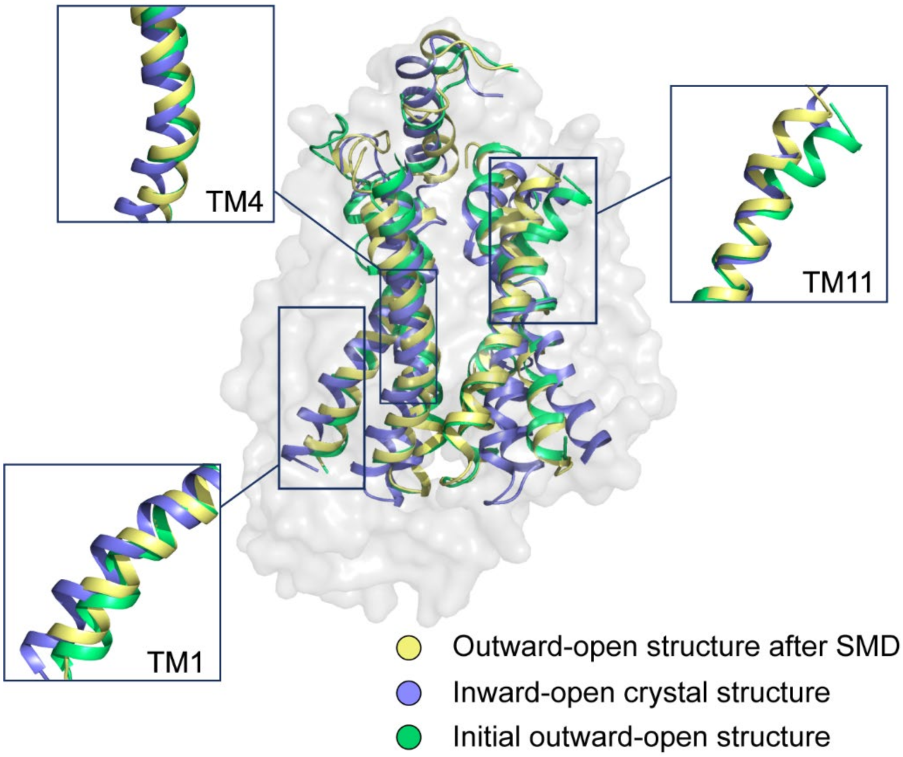

Figure S1. Structural comparison of different states of GLUT1.

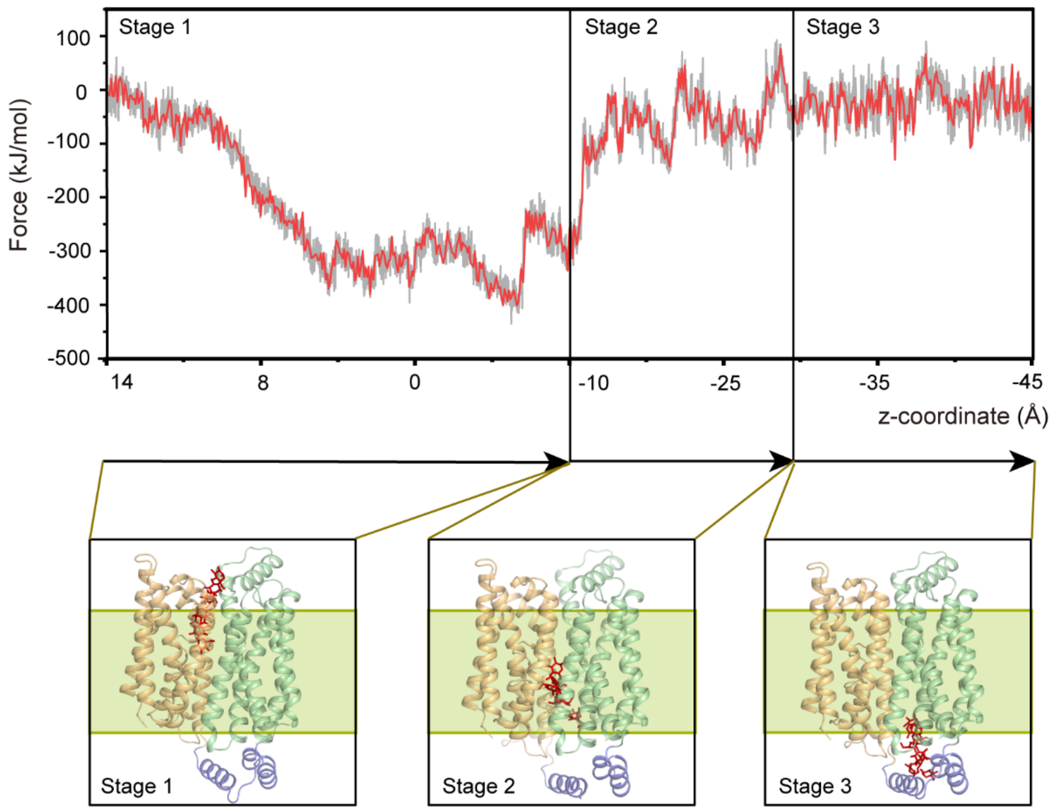

Figure S2. Force constant of the harmonic potential in the steered molecular dynamics simulations with an inwardly opening model of GLUT1. Lower force constants indicate that the glucose need to overcome higher energy barrier for its translocation.
